# A High-Throughput Screen Identifies a New Natural Product with Broad-Spectrum Antibacterial Activity

**DOI:** 10.1371/journal.pone.0031307

**Published:** 2012-02-16

**Authors:** Patrick Ymele-Leki, Shugeng Cao, Jared Sharp, Kathleen G. Lambert, Alexander J. McAdam, Robert N. Husson, Giselle Tamayo, Jon Clardy, Paula I. Watnick

**Affiliations:** 1 Division of Infectious Diseases, Children's Hospital, Boston, Boston, Massachusetts, United States of America; 2 Department of Biological Chemistry and Molecular Pharmacology, Harvard Medical School, 240 Longwood Avenue, Boston, Massachusetts, United States of America; 3 Department of Laboratory Medicine, Children's Hospital, Boston, Boston, Massachusetts, United States of America; 4 Unidad Estrategica de Bioprospeccion, Instituto Nacional de Biodiversidad (INBio) and Escuela de Química, Universidad de Costa Rica, San José, Costa Rica; University of Hyderabad, India

## Abstract

Due to the inexorable invasion of our hospitals and communities by drug-resistant bacteria, there is a pressing need for novel antibacterial agents. Here we report the development of a sensitive and robust but low-tech and inexpensive high-throughput metabolic screen for novel antibiotics. This screen is based on a colorimetric assay of pH that identifies inhibitors of bacterial sugar fermentation. After validation of the method, we screened over 39,000 crude extracts derived from organisms that grow in the diverse ecosystems of Costa Rica and identified 49 with reproducible antibacterial effects. An extract from an endophytic fungus was further characterized, and this led to the discovery of three novel natural products. One of these, which we named mirandamycin, has broad-spectrum antibacterial activity against *Escherichia coli*, *Pseudomonas aeruginosa*, *Vibrio cholerae*, methicillin-resistant *Staphylococcus aureus*, and *Mycobacterium tuberculosis*. This demonstrates the power of simple high throughput screens for rapid identification of new antibacterial agents from environmental samples.

## Introduction

Microbes that live together in the environment develop long-lasting methods to keep each other at bay. As a result, many of our most effective bactericidal agents have come from environmental organisms. These include glycopeptides such as vancomycin first isolated in 1953 [1], β-lactam derivatives such as penicillin first isolated in 1929, and aminoglycosides [2], [3].

The emergence of bacteria with resistance to multiple antimicrobial agents has motivated the development of high throughput chemical screens (HTS) to identify novel antibiotics. These screens differ in the number of samples that can reasonably be evaluated and the level of technology required to carry out the screen [4]–[7]. Furthermore, some screening assays assess inhibition of a known, purified bacterial target, while others measure toxicity to intact bacteria. The advantage of the former approach is that the target of inhibition is known for any identified compound. The great disadvantage, however, is that, in secondary screens, the compound is often found to have no activity against intact bacteria due to inadequate penetration, rapid efflux, or inactivation by bacterial products [8]. For this reason, compounds discovered in screens using whole cells are often farther along the path to the development of a successful antibacterial agent.

Here, we describe a sensitive and robust colorimetric whole cell-based HTS for antibacterial compounds. We used this assay to screen a collection of over 39,000 crude extracts from organisms that grow in the diverse ecosystems of Costa Rica. Forty-nine antibacterial extracts were identified, and, as proof of principle, one was further fractionated, leading to the elucidation of three novel natural products. One of these was found to have activity against the acid-fast bacterium, *Mycobacterium tuberculosis*, the Gram-positive bacterium, methicillin-resistant *Staphylococcus aureus* (MRSA), and several Gram-negative bacteria. Our results demonstrate the utility of simple metabolic screens in rapid identification of novel, broad-spectrum antimicrobial agents.

## Methods

### Bacterial strains and media

A *V. cholerae* O139 strain MO10 (PW357) was used for screening [9]. As a control, we used a *V. cholerae* phosphoenolpyruvate phosphotransferase (PTS) mutant (ΔEI, PW961), which is unable to transport sucrose [10]. *Mycobacterium tuberculosis* H37Rv (ATCC 27294), *Escherichia coli* (ATCC 25922), carbapenemase-positive *Klebsiella pneumonia* (ATCC BAA-1705), and methicillin-resistant *Staphylococcus aureus* (MRSA, ATCC BAA-976) were used for further evaluation of antibacterial activity.

A previously described minimal medium (MM) supplemented with sucrose (0.5% wt/vol), thymol blue (0.006% wt/vol) and bromothymol blue (0.006% wt/vol) (pH-MM^Suc^) was used for the HTS [10]. In secondary screens, MM was also supplemented with glucose (0.5% wt/vol), thymol blue (0.006% wt/vol), and bromothymol blue (0.006% wt/vol) (pH-MM^Glu^) or pyruvate (0.5% wt/vol) (MM^Pyr^).

*M. tuberculosis* H37Rv was grown at 37°C in Middlebrook 7H9 liquid medium (Difco) supplemented with albumin (0.5% wt/vol), dextrose (10 mM), glycerol (0.2% vol/vol) and Tween 80 (0.05% vol/vol) (7H9-TW80-ADC).

### Fungal culture

Agar plugs containing the endophytic fungal isolate 1223-D were initially grown at 25°C on yeast malt agar plates supplemented with streptomycin (30 µg/mL) and chlortetracycline (12 µg/mL). After one week, 3 macerated agar plugs were placed in 75 mL of rich seed media consisting of peptone (5 g/L), dextrose (10 g/L), yeast extract (3 g/L), and malt extract (10 g/L) adjusted to pH 6.2 and cultured at 25°C with shaking for 6 days. 450 mL of malt extract (0.66% wt/vol) and 10 g HP-20 resin were then added to each flask, and the fungi were cultured under the same conditions for 21 days. The fungal culture was subsequently incubated statically at 25°C for 5 days and filtered. The HP-20 resin with mycelia was extracted three times with 200 mL of ethanol to yield the crude extract.

### Natural product library

The natural product library, which was prepared in Costa Rica (collection permits 307-2003-OFAU, R-CM-03-2006, R-CM-INBio-06-2006, R-CM-INBio-082-2009, R-CM-INBio-04-2009, R-CM-INBio-088-2009 and R-CM-INBio-094-2010), consisted mainly of pre-fractionated extracts from microbial sources, such as fungal endophytes and marine bacteria, although extracts from other sources such as marine invertebrates, cyanobacteria and lichens were also included [11]. Extracts were suspended in dimethyl sulfoxide (DMSO) at a concentration of ∼15 mg/mL. The compound library was stored at −20°C in dessicated storage containers.

### HTS for antimicrobial activity

**Figure 1:**
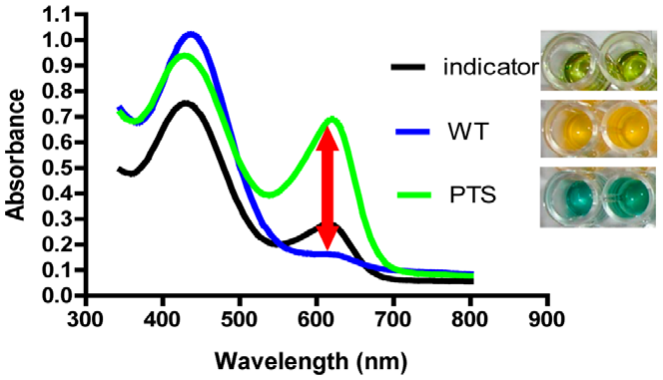
Flow chart of HTS assay. The HTS assay begins with manual preparation of working solutions of pH-MM^Suc^ and a bacterial suspension with OD_600_ of 0.015 in PBS. Subsequent steps of the assay are fully automated: solution mixing in the 384 well-plates is performed by a ThermoScientific Matrix WellMate liquid dispenser, pin-transfer of the natural extracts tested is done with a custom-built Epson robot, and A_615_ readout after incubation at room temperature is accomplished at 6 and 20 hours using an EnVision™ multi-well spectrophotometer. Finally, EnVision™ data were analyzed with Spotfire™ and Excel. Each assay is performed in duplicate. A measurement was considered to be statistically significant if it deviated by at least three standard deviations from the mean measurement calculated using all measurements made with a particular extract library. The calculated Z′ factor for the screen was 0.808±0.088.

The first step of compound identification was an HTS for inhibitors of *V. cholerae* sucrose fermentation in pH-MM^Suc^ medium. A work-flow chart for this HTS is shown in Figure 1. Fermentation decreases the pH of the medium. pH indicators in the medium allowed us to monitor medium acidification spectrophotometrically through a change in absorbance at 615 nm (A_615_). To initiate the assay, *V. cholerae* derived from a glycerol stock was streaked on an LB-agar plate and incubated overnight at 37°C. A loopful of cells was harvested, washed three times with PBS, and then resuspended in PBS at an optical density of 0.015. For the HTS, 10 µL of this bacterial cell suspension was aliquoted into the wells of a 384-well plate containing 30 µL of pH-MM^Suc^ and 100 nL of the test compound. For each assay, the A_615_ was measured after incubation at room temperature for 6 and 20 hours. This step was automated and validated in 384-well plate format using an EnVision™ multi-well spectrophotometer.

### Compound isolation and identification

The crude extract was resuspended in 90% water/methanol and passed over a C18 SPE column to get fraction I. The column was then washed with methanol to get fraction II. The compound mixture in fraction II was separated on an Agilent 1100 series HPLC with a preparative Phenyl-hexyl column (Phenomenex, Luna, 25 cm×10 mm, 5 µm particle size) using an elution buffer containing 20% acetonitrile/water with 0.1% formic acid at a flow rate of 2 mL/min for 50 minutes. This yielded compound 1 (*t_R_*: 23.5 min), compound 2 (*t_R_*: 25 min), and compound 3 (*t_R_*: 44 min). Spectra for compound identification were obtained on an Alpha FT-IR mass spectrometer (Bruker), an Ultrospec™ 5300 *pro* UV/Visible Spectrophotometer (Amersham Biosciences), and an INOVA 600 MHz nuclear magnetic resonance spectrometer (Varian).

### Determination of minimum inhibitory concentrations (MIC)

The MICs for all species except for *M. tuberculosis* were determined in cation-adjusted Mueller-Hinton broth (CAMHB) using the microdilution broth method, according to M07-A8 and M100-S21 guidelines [12], [13]. Standardized inocula of each bacterium were prepared from cultures grown overnight at 37°C in CAMHB, which were subsequently diluted 1∶50 in fresh CAMHB and grown for 3 h at 37°C without shaking. Each log-phase culture was diluted to deliver a final bacterial density of 5×10^5^ CFU per mL. To perform the tests, a dilution series of the indicated antimicrobial agent in CAMHB was prepared from a stock solution containing 10 mg/mL of the compound in DMSO. The final concentrations of the natural product were between 0.625 and 80 µg/mL. These were chosen because we knew that the compound was active against *V. cholerae* within this range. Dilutions of known antimicrobial compounds were similarly chosen based on the reported MIC's for the bacterium in question. An MIC 2000 inoculator (DynaTech) was used to accurately dispense 1.5 µL of bacterial culture into 100 µL of CAMHB alone or supplemented with an antimicrobial agent in a 96 well plate. The plates were prepared in duplicate and incubated overnight at 37°C. A positive control for growth containing no antimicrobial compound but the relevant amount of DMSO and a negative growth control containing no bacteria were also prepared for each assay. The MIC was determined visually as the lowest antimicrobial agent concentration that prevented bacterial growth. The *M. tuberculosis* MIC was determined by the fluorometric microplate-based Alamar blue assay (MABA) in 7H9 liquid media containing casein (0.1% wt/vol) and lacking Tween 80 (7H9-Casein-ADC) [14], [15].

### Microdilution Alamar blue assay for *M. tuberculosis*

Briefly, 1 mL of *M. tuberculosis* cell stock was added to 49 mL of 7H9-TW80-ADC media and incubated for 4–5 days at 37°C with shaking (120 rpm) until an OD_600_ of 0.6–0.8 (#3–4 McFarland turbidity standard) was reached. The cells were then washed twice with PBS and resuspended in 7H9-Casein-ADC to a final concentration of 2×10^5^ cells/mL. 100 µl of the cell suspension was inoculated into the wells of clear-bottomed, 96-well microplates preloaded with 100 µl of 7H9-Casein-ADC media containing appropriate dilutions of the test compound. Initial compound dilutions were prepared in DMSO, and subsequent two-fold dilutions were directly performed in the microtiter plates used for the assay. To determine if bacterial densities were adequate for the assay, 32.5 µl of Alamar blue solution (10×Alamar blue dye, 20% Tween 80, 8×PBS, pH 7) were added to a control well after 6–7 days of growth at 37°C. If the control well remained blue or turned purple and/or had a fluorescence reading <17,500 fluorescence units (FU) after 18–24 hours of further growth at 37°C, additional control wells were tested daily until the well turned pink and the fluorescence reading was greater than 17,500 FU's. At this point, the Alamar blue solution was added to the entire plate, and the fluorescence was measured after overnight incubation. All fluorescence measurements were performed in an HTS7000 Plus Bio Assay Reader (Perkin Elmer) in bottom-reading mode with excitation at 550 nm and emission at 595 nm. Percent inhibition was defined as (experimental well FU – media only FU)/(bacteria only FU – media only FU)×100. The lowest drug concentration effecting ≥90% inhibition was considered the MIC.

### Fungal identification by internal transcribed spacer amplification (ITS) and sequencing

For fungal identification, isolate 1223-D was cultured on agar as described above for 26 days. The mycelium was then retrieved and ground to a fine powder in liquid nitrogen. Genomic DNA was extracted using the Wizard Genomic DNA Purification Kit (Promega), and the large subunit ribosomal DNA was amplified by PCR using primers LR5 (5′-TCCTGAGGGAAACTTCG-3′) and LROR (5′-ACCCGCTGAACTTAAGC-3′) as well as their reverse complements. The PCR products were submitted for sequence analysis (Genewiz), and the resulting sequences were used in a BLAST search against deposited sequences. These sequences are shown in Text S1.

## Results

### Development of a high throughput screen

In the clinical microbiology laboratory, one of the characteristics used to distinguish *V. cholerae* from other *Vibrio* species is its ability to ferment sucrose on thiosulfate-citrate-bile salts-sucrose (TCBS) plates, which contain the pH indicators bromothymol blue and thymol blue. As the pH of a solution decreases below 7.1 for bromothymol blue and 8.0 for thymol blue, these aromatic compounds, which are weak acids, gain a proton resulting in a color change from blue to yellow. This process is reversible. Therefore, if the pH is increased again, the color of these weak acids will return to blue. Such pH indicators are often used as reporters of bacterial fermentation [16]–[18].

We recently showed that transport of sucrose by *V. cholerae* depends entirely on a phosphotransfer cascade known as the phosphoenolpyruvate phosphotransferase system or PTS, which also regulates biofilm formation [10], [19]. We were interested in developing a reporter medium that would allow us to identify compounds that inhibit transport through the PTS, sugar fermentation, or bacterial growth. Therefore, we added bromothymol blue and thymol blue, the pH indicators found in TCBS agar, to minimal medium containing sucrose (pH-MM^Suc^). Based on the pKa values of bromothymol blue and thymol blue, we predicted that the medium would be yellow at pH<7.1, when both indicators are protonated. We predicted that the medium would be green at a pH between 7.1 and 8.0 because bromothymol blue would be blue due to deprotonation, while thymol blue would remain yellow. At pH>8.0, when both indicators are deprotonated, we anticipated that the medium would be blue.

**Figure 2:**
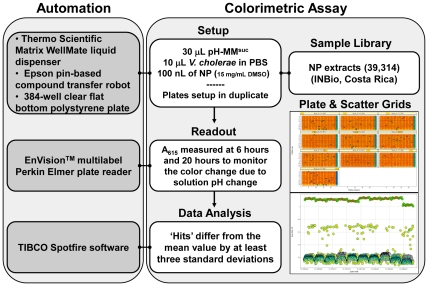
Spectrophotometric assay for bacterial sugar fermentation. Absorbance spectrum of MM^Suc^ alone (indicator) or incubated with wild-type *V. cholerae* (WT) or a PTS mutant for 5 hours. The spectra are shown at the left, while the visible color difference is shown in microtiter dish wells at the right. The largest difference in absorbance between MM^Suc^ incubated with wild-type *V. cholerae* and that incubated with a PTS mutant is measured at 615 nm (red arrow).

Incubation of wild-type *V. cholerae* in this medium caused a change in color from green to yellow due to fermentation (Figure 2). When the PTS mutant, which cannot utilize sucrose, was incubated in pH-MM^Suc^, the medium turned blue, indicating an increase in the pH. This is the result of amino acid catabolism (Figure 2), which generates ammonia, a weak base. We hypothesized that these visible differences in the color of the medium at low and high pH were the result of a change in absorbance at a wavelength in the visible range. To identify this wavelength, we scanned the visible spectra of pH-MM^Suc^ alone, pH-MM^Suc^ incubated with wild-type bacteria, and pH-MM^Suc^ incubated with a PTS mutant. As shown in Figure 2, the maximum difference in absorbance for all these conditions was observed at a wavelength of 615 nm. We based our screen on this observation. It consisted of a room temperature incubation of *V. cholerae* in MM^Suc^ supplemented with bromothymol blue and thymol blue, and measurements of A_615_ at 6 and 20 hrs (Figure 1).

To validate the screen, a pilot assay was conducted in 384 well microtiter dishes using plates 1568 and 1569 from the Prestwick Collection, a commercial library. Wild-type *V. cholerae* with no added compound was used as a positive control, while a PTS mutant, which is unable to transport sucrose, was used as a negative control. One column of each dish was reserved for replicate positive controls and another for replicate negative controls. To evaluate the performance of our assay, we calculated a Z′ factor for each microtiter dish. This factor reflects the difference between positive and negative control measurements [20] and is used to determine whether the size of a response (e.g. change in absorbance) is large enough to be useful in a HTS. A Z′ factor between 1 and 0.9 is considered excellent, while one between 0.9 and 0.7 is considered good. For these tests, the Z′ factor ranged from 0.785 to 0.914, suggesting that this was a good to excellent assay. Therefore, we proceeded with the HTS.

### HTS of natural products

We carried out a screen of a library of partially purified extracts from diverse Costa Rican organisms. The library consisted of 39,314 extracts arrayed in 384-well plates at a concentration of ∼15 mg/mL in DMSO (see: http://iccb.med.harvard.edu/screening/compound_libraries/index.htm#natural). Each library plate contained extracts in columns 1–22 and DMSO only in columns 23 and 24. Compounds were pin-transferred into a 384-well plate pre-filled with pH-MM^Suc^. Columns 1–23 were then inoculated with wild-type *V. cholerae*, and column 24 was inoculated with a PTS mutant. Columns 23 and 24 served as fermentation-positive and fermentation-negative controls, respectively. Each plate of extracts was tested in duplicate. A measurement was considered to be both statistically and biologically significant if it deviated by at least three standard deviations from the mean measurement, which was calculated from measurements derived from all the compounds screened. Statistical analysis performed after completion of the screen yielded a Z factor with a mean value of 0.808±0.088, indicating a very robust screen. We identified 126 unique extracts with possible activity against *V. cholerae*.

### Secondary screens

Compounds that increase the pH of the medium or that absorb in the visible spectrum could be a source of false positives in this assay. These were easily eliminated by detailed monitoring of the change in A_615_ over time. In addition, the following secondary screens were designed to identify (i) inhibitors of PTS sugar transport, (ii) inhibitors of sugar fermentation, or (iii) inhibitors of bacterial growth. To distinguish between extracts that inhibited PTS-dependent sugar transport and those that delayed fermentation, we compared medium acidification in MM^Glu^ with that in MM^Suc^ in the presence of crude extracts with the following rationale. After hydrolysis, the fermentation pathway of sucrose is similar to that of glucose. However, unlike sucrose, glucose is transported by both PTS-dependent and PTS-independent means [10]. Therefore, we predicted that, in the presence of specific inhibitors of the PTS, medium acidification would proceed more slowly in MM^Suc^ than in MM^Glu^. In contrast, inhibitors of fermentation should behave similarly in both media. Because pyruvate is transported independently of the PTS and is not fermented, we used growth in MM^Pyr^ to identify extracts that inhibited bacterial replication. Each assay was performed in duplicate, and each reported value represents the average of two experimental replicates. To account for the variability of initial absorbance measurements, experimental data were normalized to the initial A_615_ for each well.

**Figure 3:**
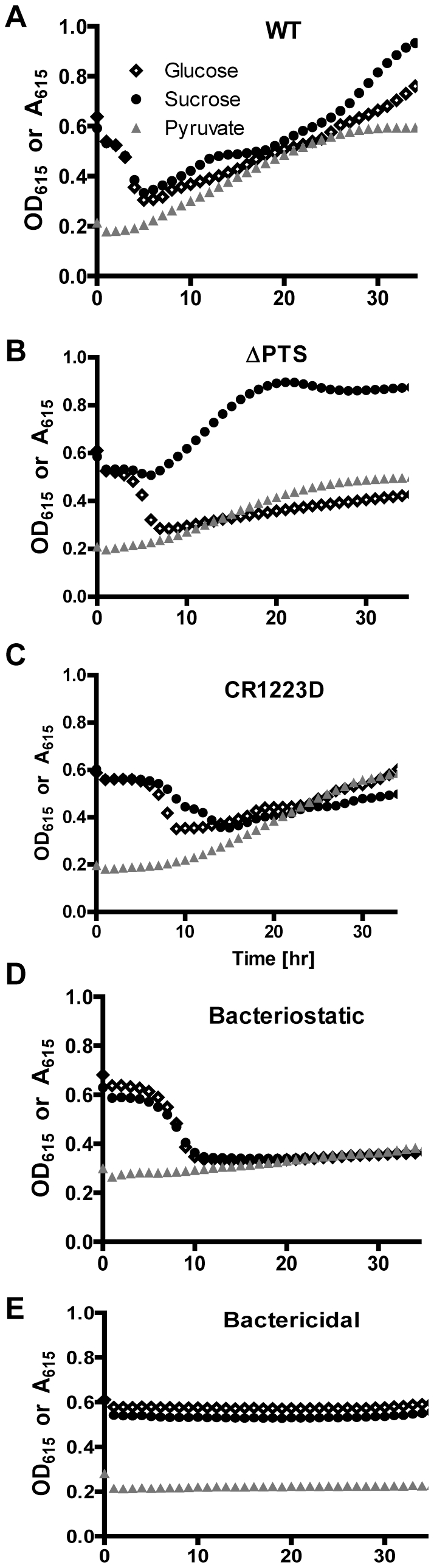
Representative results for secondary screen. Bacteria were grown in MM^Pyr^, pH-MM^Suc^, or pH-MM^Glu^. OD_615_ measurements of cultures in MM^Pyr^ reflect the ability of cells to grow in the presence of extract, while absorbance measurements in pH-MM^Suc^ and pH-MM^Glu^ reflect the ability of cells to transport and ferment these sugars in the presence of extract. Data are shown for wild-type *V. cholerae* and a PTS mutant in the absence of extract (A,B) or for wild-type *V. cholere* in the presence of extracts that we hypothesize (C) interfere with sugar transport and fermentation, (D) inhibit bacterial growth (bacteriostatic), or (E) kill bacteria (bactericidal).

We first compared the performance of wild-type *V. cholerae* and a PTS mutant in our proposed secondary screens (Figure 3A and B). In MM^Suc^ and MM^Glu^ containing wild-type *V. cholerae* alone (Figure 3A), the A_615_ initially decreased but then began to rise after approximately 5 hours of incubation. We hypothesized that the initial decrease in A_615_ represented acidification of the medium due to sugar fermentation, while the subsequent increase in A_615_ reflected depletion of the sugar supply and initiation of amino acid catabolism as well as cell growth.

An increase in A_615_ was observed during incubation of the PTS mutant in MM^Suc^ due to its inability to transport and consequently ferment sucrose (Figure 3B). Unlike sucrose, glucose can be transported by the PTS mutant. Therefore, in MM^Glu^, the A_615_ of the medium initially decreased, albeit more slowly than was observed for incubation with wild-type *V. cholerae*. Lastly, wild-type *V. cholerae* and the PTS mutant grew equally well in MM^Pyr^.

Our secondary screen yielded 49 extracts with reproducible effects on medium acidification by *V. cholerae* (see Table S1 and Figure S1). These included (i) one extract, CR1223-D, which delayed medium acidification by sucrose fermentation more than that by glucose fermentation, (ii) 34 extracts that blocked growth in pyruvate but not medium acidification, and (iii) fourteen extracts that blocked growth in pyruvate as well as medium acidification (representative traces are shown in Figure 3C–E). We hypothesized that CR1223-D might contain an inhibitor of PTS transport. Furthermore, we reasoned that medium acidification in the absence of cell growth, as was seen in group (ii), reflected the presence of viable bacteria whose growth was inhibited. Therefore, we hypothesized that these extracts were bacteriostatic. The absence of both medium acidification and cell growth, as was observed in group (iii), suggested the absence of viable bacteria. We hypothesized that these extracts were bactericidal, although it is formally possible that these extracts contained compounds that inhibited both sugar transport and cell growth while preserving the viability of bacterial cells.

Because of our interest in sugar metabolism, we subsequently focused on characterization of CR1223-D. This extract was derived from isolate 1223-D, an unclassified endophytic fungus harvested from the twig of *Neomirandea angularis*, a host plant from the Asteraceae family. Amplification, sequencing (see SI), and alignment of the ITS region using Mega [21] suggested that this fungus was most closely related to the environmental fungi, *Septofusidium herbarum* and *Acremonium alternatum.*

### Isolation and identification of three novel natural products from isolate 1223-D

**Figure 4:**
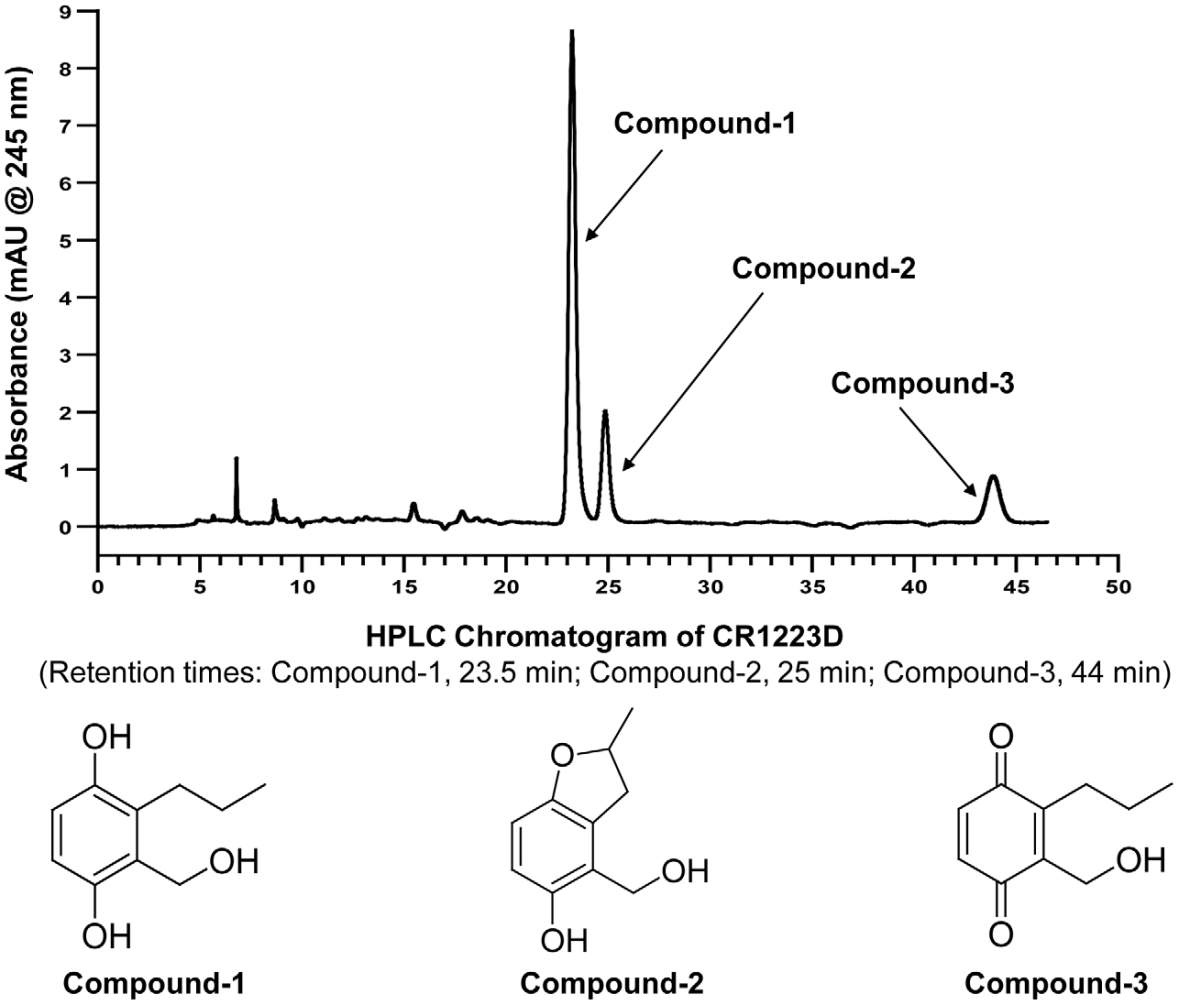
Isolation and identification of three novel natural compounds. Chromatogram obtained during fractionation of the crude extract CR1223-D showing three peaks corresponding to the three compounds isolated. Compound 1 was further identified as 6-propyl gentisyl alcohol, compound 2 as 5-hydroxy-4-(hydroxymethyl)-2-methyl 2,3-dihydrobenzofuran, and compound 3 as 2-(hydroxymethyl)-3-propyl benzoquinone.

Extracts of fungal isolate1223-D were prepared and fractionated as described in the Materials and Methods. High resolution mass spectrometry, infrared and ultraviolet spectrometry, and ^1^H and ^13^C nuclear magnetic resonance spectroscopy were used to identify compound 1 as 6-propyl gentisyl alcohol or 2-(hydroxymethyl)-3-propyl hydroquinone, compound 2 as 5-hydroxy-4-(hydroxymethyl)-2-methyl 2,3-dihydrobenzofuran, and compound 3 as 2-(hydroxymethyl)-3-propyl benzoquinone (Figure 4). The physical properties and NMR spectra of these compounds are described in detail in Text S2 and Table S2. These are all previously unreported natural products.

### Activity of compounds 1, 2, and 3 against *V. cholerae*

**Figure 5:**
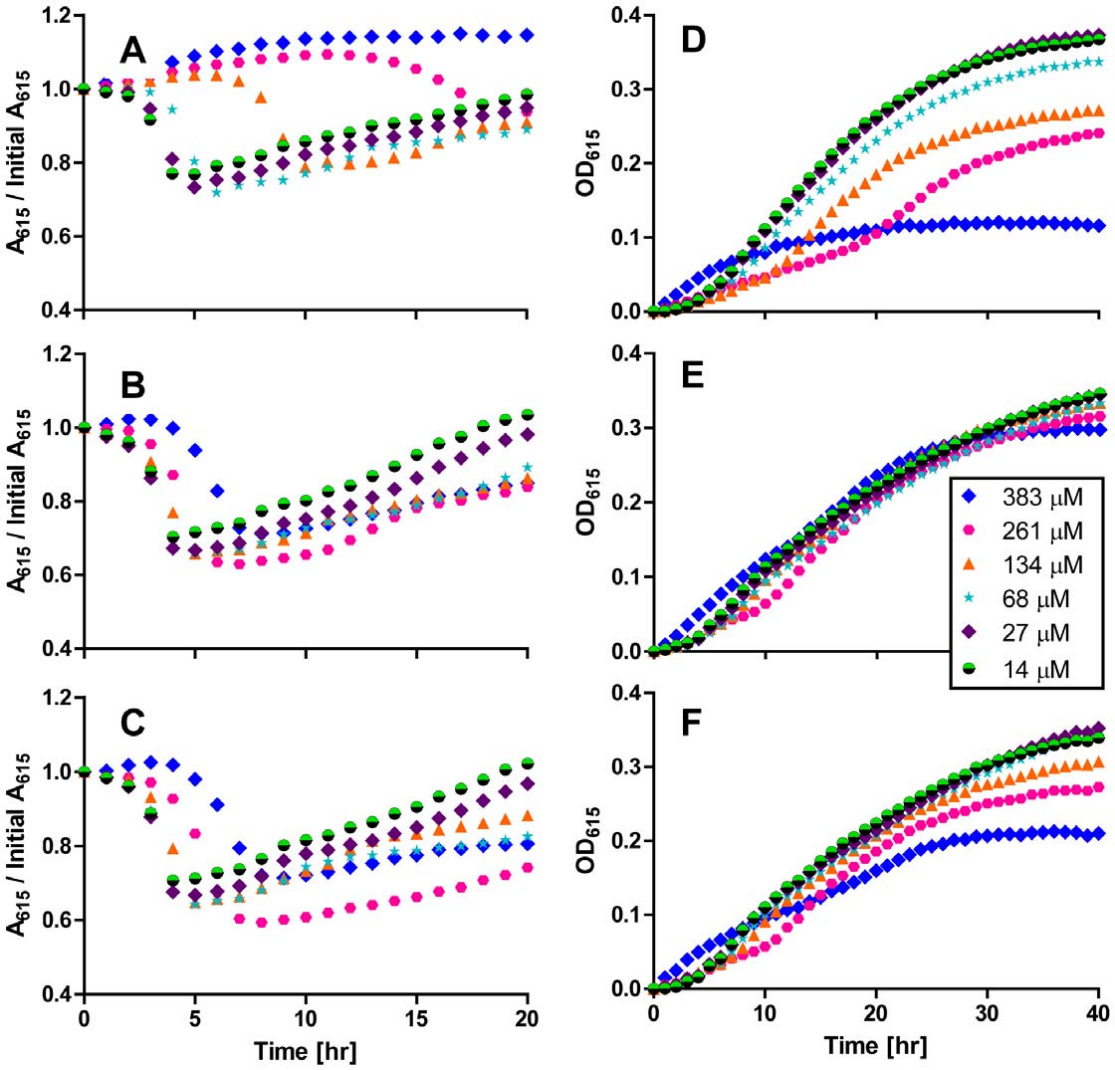
Impact of compounds 1, 2, and 3 on *V. cholerae* sugar fermentation and growth. The assays were carried out at 30°C in pH-MM^Suc^ to monitor sugar fermentation by A_615_ (A, B, and C) or in MM^Pyr^ to monitor bacterial growth by OD_615_ (D, E, and F). Bacteria were exposed to mirandamycin (A and D), compound 2 (B and E) or compound 3 (C and F) at concentrations ranging from 14 to 383 µM. Replicate assay is shown in Figure S2.

To determine which of these compound(s) was responsible for the activity of CR1223-D, we performed medium acidification and growth assays in the presence of various concentrations of compounds 1 through 3. Conditions were tested in duplicate in each experiment, and two experimental replicates were performed on separate days. Reproducibility was excellent. The result of one experiment is shown in Figure 5, while the result of the replicate experiment is shown in Figure S2. Compounds 2 and 3 had only modest effects on medium acidification and growth even at the highest concentrations studied. In contrast, compound 1 inhibited medium acidification at a concentration of 134 µM and completely blocked medium acidification at a concentration of 261 µM. However, only modest effects on growth were observed at these concentrations. At higher concentrations, compound 1 was able to completely inhibit growth of *V. cholerae*. To determine whether this represented bacteriostatic or bactericidal activity, dilutions of the cell suspensions were plated on LB agar after 20 hours of growth in MM^Pyr^ supplemented with compound 1 at a concentration of 383 µM. No CFU were documented after 24 hours of incubation at 37°C, indicating that compound 1 inhibited fermentation and possibly sugar transport at lower concentrations and was bactericidal at higher concentrations. Thus, we concluded that compound 1 was responsible for the inhibitory activity of CR1223-D detected in our HTS assay. We have named compound 1 mirandamycin, after the genus of the host plant of the producing fungus.

### *In vitro* antimicrobial activity of mirandamycin against other bacterial pathogens

To evaluate the antimicrobial efficacy of mirandamycin against a broader panel of bacterial pathogens, we measured the activity of compound 1 against clinical strains of *E. coli*, *P. aeruginosa*, carbapenemase-producing *K. pneumonia*, methicillin-resistant *S. aureus*, and *M. tuberculosis*. As shown in Table 1, mirandamycin was most active against Gram-positive organisms but also had some activity against the more sensitive Gram-negative rods. Susceptibility of these organisms to known antibiotics is shown for comparison.

**Table 1:** Minimum inhibitory concentrations (MICs) of mirandamycin and known antibiotics against selected bacterial pathogens.

Species	ATCC	MIC (µg/mL)				
		MIR	LEVO	AMP	IMI	TMP/SMX
*E. coli*	25922	80	0.019	2.5	1.25	0.125
*P. aeruginosa*	27853	80	1.25	>80	5	>16
*K. pneumoniae* carbapenemase positive	BAA-1705	>80	>80	>80	>80	>16
MRSA	BAA-976	10	0.312	>80	1.25	0.062
*V. cholerae* PW357	-	40	<0.005	2.5	1.25	>16

		MIR	LEVO	INH	PZA	ETH
*M. tuberculosis* H37Rv (*)	27294	25	0.25	0.25	100	2

(*) MIC was determined by Alamar Blue Assay as described in Material and Methods; Mirandamycin (MIR), levofloxacin (LEVO), ampicillin (AMP), imipenem (IMI), bactrim (TMP/SMX), isoniazid (INH), pyrazinamide (PZA), ethambutol: ETH.

## Discussion

We have developed and implemented a simple, inexpensive, and robust HTS for antibacterial agents based on a spectrophotometric assay of sugar fermentation, a process present only in viable bacteria. Secondary screens allowed us to easily distinguish between bactericidal and bacteriostatic compounds as well as those that blocked sugar fermentation but did not decrease growth or viability.

As compared with other HTS for antibacterial compounds, this screen has several advantages. First of all, the screen uses whole cells rather than purified targets [7]. Secondly, it is an assay for cell viability and, therefore, is biased toward bactericidal agents [22], [23]. Thirdly, because it does not require cell growth, it is rapid. Lastly, the screen does not require expensive fluorescent reporters of cell viability.

As a proof of principle, we identified several extracts with anti-bacterial activity. Fractionation of one of these derived from an endophytic fungus led to the identification of three novel natural products. One of these natural products, a hydroquinone that we have called mirandamycin, has antibacterial activity against a wide range of difficult to treat pathogens including *P. aeruginosa*, MRSA, and *M. tuberculosis*.

Quinones and their corresponding reduced forms, the hydroquinones, are components of eukaryotic and bacterial electron transport chains. In *V. cholerae*, ubiquinone-8 is reduced by the Na+-translocating NADH∶ubiquinone oxidoreductase (NQR) at the cytoplasmic face of the inner membrane. The corresponding hydroquinone then diffuses across the inner membrane where it is oxidized by one of several possible quinol oxidases, discharging protons to the periplasmic space. The resulting quinone is recycled to the inner membrane [24]. Therefore, quinones are reduced at the cytoplasmic face of the inner membrane and the corresponding hydroquinones are oxidized at the periplasmic face.

The bactericidal secondary metabolite identified here, mirandamycin, is a hydroquinone, closely related to homogentisic acid. We hypothesize that the antibacterial activity of mirandamycin is the result of an interaction with an outward facing bacterial quinol oxidase. One possibility is that single electron oxidation of mirandamycin by a quinol oxidase results in formation of a semiquinone intermediate, which can then react with molecular oxygen to produce a toxic superoxide radical. Inhibition of bacterial quinol oxidase by mirandamycin is another possible antibacterial mechanism.

Quinones are known to be toxic to both mammalian and bacterial cells [25], [26]. First of all, they can undergo single electron reduction at the cytoplasmic face of bacterial cell membranes to form semiquinones. Secondly, quinones can interact with thiol-containing compounds to form adducts. Interestingly, the oxidized quinone form of mirandamycin reported here (compound 3) demonstrated no antibacterial activity. We hypothesize that compound 3 does not enter bacterial cells and, therefore, is not reduced to mirandamycin under the conditions of our experiment.

Organisms that survive successfully in close proximity to bacterial pathogens have been a rich source of potent antibacterial natural products. Here we present an easily implemented, sensitive HTS that rapidly identified a large number of antibacterial extracts from environmental samples. Through this screen, we identified a quinol with activity against multiple pathogens. In this era of rising resistance to existing antibiotics, approaches such as this will be increasingly relied on to fill our antimicrobial pipeline.

## Supporting Information

Figure S1 **Natural extracts with reproducible effects on medium acidification by** ***V. cholerae*** **.** Time course measurements of *V. cholerae* medium acidification in pH-MM^Suc^ and pH-MM^Glu^ or growth in MM^Pyr^ either alone (boxed trace) or in the presence of test extracts. Extract designation is indicated above each trace (see Table S1 for additional information).
(PDF)

Figure S2 **Impact of compounds 1, 2, and 3 on** ***V. cholerae*** **sugar fermentation and growth.** Experimental replicate of Figure 5.
(PDF)

Table S1 **Natural extracts with reproducible effects on medium acidification by** ***V. cholerae.***
(PDF)

Table S2 **^1^H and ^13^C NMR data of compounds 1 to 3.**
(PDF)

Text S1 **Sequence analysis of products obtained from amplification of the internal transcribed spacer of isolate 1223-D.**
(PDF)

Text S2 **Physical characterization of compounds 1, 2, and 3 derived from CR1223-D.**
(PDF)

## References

[1] Levine DP (2006). Vancomycin: a history.. Clin Infect Dis.

[2] Fernandes P (2006). Antibacterial discovery and development–the failure of success?. Nat Biotechnol.

[3] Kohanski MA, Dwyer DJ, Collins JJ (2010). How antibiotics kill bacteria: from targets to networks.. Nat Rev Microbiol.

[4] Mohamad S, Zin NM, Wahab HA, Ibrahim P, Sulaiman SF (2011). Antituberculosis potential of some ethnobotanically selected Malaysian plants.. J Ethnopharmacol.

[5] Muh U, Schuster M, Heim R, Singh A, Olson ER (2006). Novel *Pseudomonas aeruginosa* quorum-sensing inhibitors identified in an ultra-high-throughput screen.. Antimicrob Agents Chemother.

[6] Parish CA, de la Cruz M, Smith SK, Zink D, Baxter J (2009). Antisense-guided isolation and structure elucidation of pannomycin, a substituted cis-decalin from Geomyces pannorum.. J Nat Prod.

[7] Pereira MP, Blanchard JE, Murphy C, Roderick SL, Brown ED (2009). High-throughput screening identifies novel inhibitors of the acetyltransferase activity of *Escherichia coli* GlmU.. Antimicrob Agents Chemother.

[8] Fischbach MA, Walsh CT (2009). Antibiotics for emerging pathogens.. Science.

[9] Waldor MK, Colwell R, Mekalanos JJ (1994). The *Vibrio cholerae* O139 serogroup antigen includes an O-polysaccharide capsule and lipopolysaccharide virulence determinant.. Proc Natl Acad Sci USA.

[10] Houot L, Watnick PI (2008). A novel role for enzyme I of the *Vibrio cholerae* phosphoenolpyruvate phosphotransferase system in regulation of growth in a biofilm.. J Bacteriol.

[11] Cao S, Ross L, Tamayo G, Clardy J (2010). Asterogynins: secondary metabolites from a Costa Rican endophytic fungus.. Org Lett.

[12] Clinical and Laboratory Standards Institute (2009). Methods for dilution antimicrobial susceptibility tests for bacteria that grow aerobically; approved standard M07-A8, 8th ed..

[13] Clinical and Laboratory Standards Institute (2011). Performance standards for antimicrobial susceptibility testing, 21^st^ informational supplement M100-S21..

[14] Ananthan S, Faaleolea ER, Goldman RC, Hobrath JV, Kwong CD (2009). High-throughput screening for inhibitors of *Mycobacterium tuberculosis* H37Rv.. Tuberculosis (Edinb).

[15] Collins L, Franzblau SG (1997). Microplate alamar blue assay versus BACTEC 460 system for high-throughput screening of compounds against *Mycobacterium tuberculosis* and *Mycobacterium avium*.. Antimicrob Agents Chemother.

[16] Mandal S, Mandal MD, Pal NK (2011). Cholera: a great global concern.. Asian Pac J Trop Med.

[17] McCormack WM, DeWitt WE, Bailey PE, Morris GK, Soeharjono P (1974). Evaluation of thiosulfate-citrate-bile salts-sucrose agar, a selective medium for the isolation of *Vibrio cholerae* and other pathogenic vibrios.. J Infect Dis.

[18] Pfeffer C, Oliver JD (2003). A comparison of thiosulphate-citrate-bile salts-sucrose (TCBS) agar and thiosulphate-chloride-iodide (TCI) agar for the isolation of *Vibrio* species from estuarine environments.. Lett Appl Microbiol.

[19] Houot L, Chang S, Pickering BS, Absalon C, Watnick PI (2010). The phosphoenolpyruvate phosphotransferase system regulates *Vibrio cholerae* biofilm formation through multiple independent pathways.. J Bacteriol.

[20] Zhang JH, Chung TD, Oldenburg KR (1999). A Simple Statistical Parameter for Use in Evaluation and Validation of High Throughput Screening Assays.. J Biomol Screen.

[21] Tamura K, Peterson D, Peterson N, Stecher G, Nei M (2011). MEGA5: Molecular Evolutionary Genetics Analysis Using Maximum Likelihood, Evolutionary Distance, and Maximum Parsimony Methods.. Mol Biol Evol.

[22] Campbell J (2010). High-throughput assessment of bacterial growth inhibition by optical density measurements.. Curr Protoc Chem Biol.

[23] Ferrand S, Tao J, Shen X, McGuire D, Schmid A Screening for mevalonate biosynthetic pathway inhibitors using sensitized bacterial strains.. J Biomol Screen.

[24] Hase CC, Barquera B (2001). Role of sodium bioenergetics in *Vibrio cholerae*.. Biochim Biophys Acta.

[25] Bolton JL, Trush MA, Penning TM, Dryhurst G, Monks TJ (2000). Role of quinones in toxicology.. Chem Res Toxicol.

[26] Liebeke M, Pother DC, van Duy N, Albrecht D, Becher D (2008). Depletion of thiol-containing proteins in response to quinones in *Bacillus subtilis*.. Mol Microbiol.

