# Correction: A High-Throughput Screen Identifies a New Natural Product with Broad-Spectrum Antibacterial Activity

**DOI:** 10.1371/annotation/7efd3085-dd48-4210-9b7a-9ddb1acaa608

**Published:** 2012-04-03

**Authors:** Patrick Ymele-Leki, Shugeng Cao, Jared Sharp, Kathleen G. Lambert, Alexander J. McAdam, Robert N. Husson, Giselle Tamayo, Jon Clardy, Paula I. Watnick

There was an error in the placement in Figures 1 and 2. The figures were switched. The captions are placed correctly. 

