# Supplementary figures and images for: A High-Throughput Screen Identifies a New Natural Product with Broad-Spectrum Antibacterial Activity
Source: PLoS One. 2012 Feb 16;7(2):e31307. doi: 10.1371/journal.pone.0031307 (PMC3281070; doi:10.1371/journal.pone.0031307)

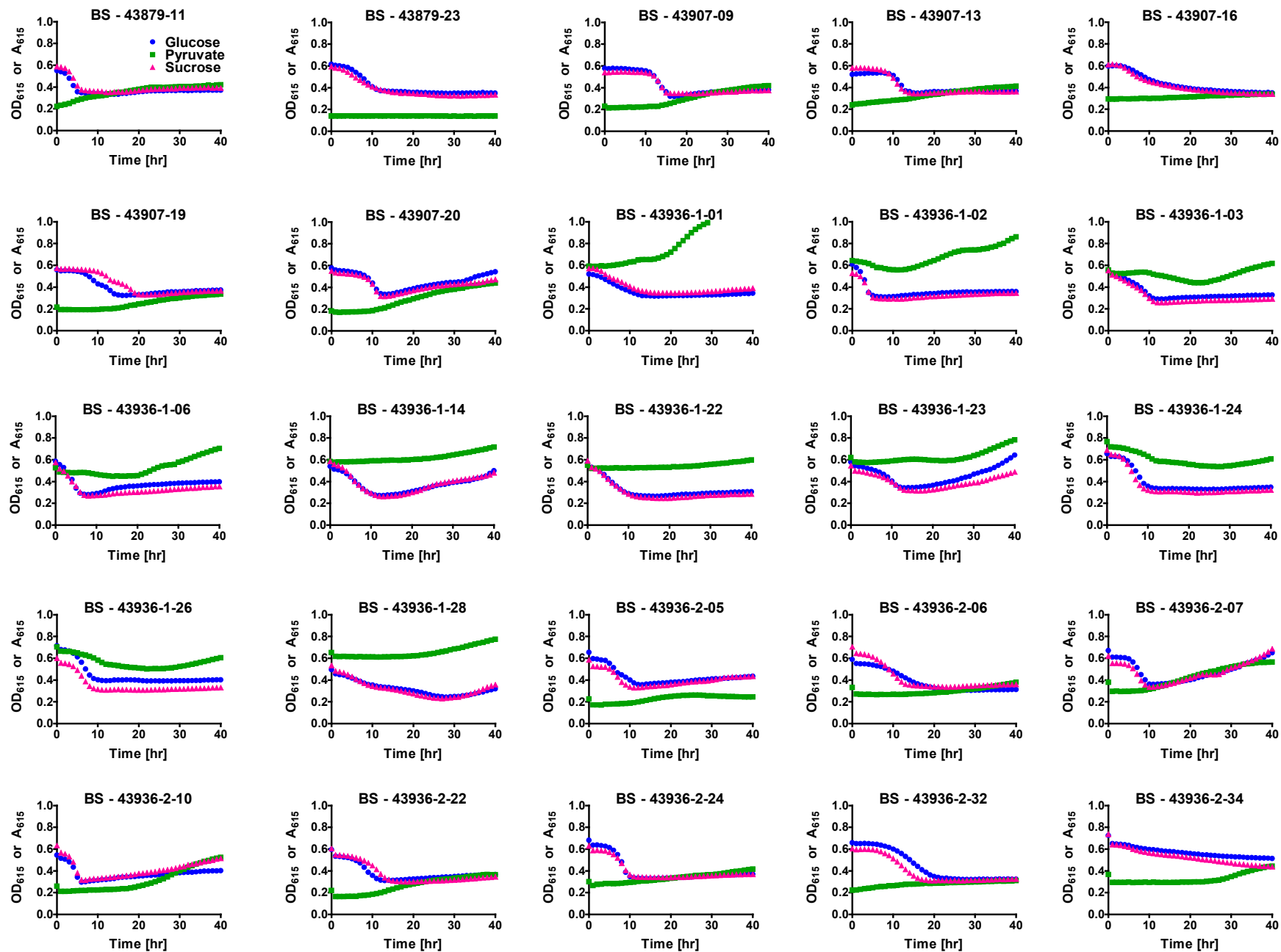

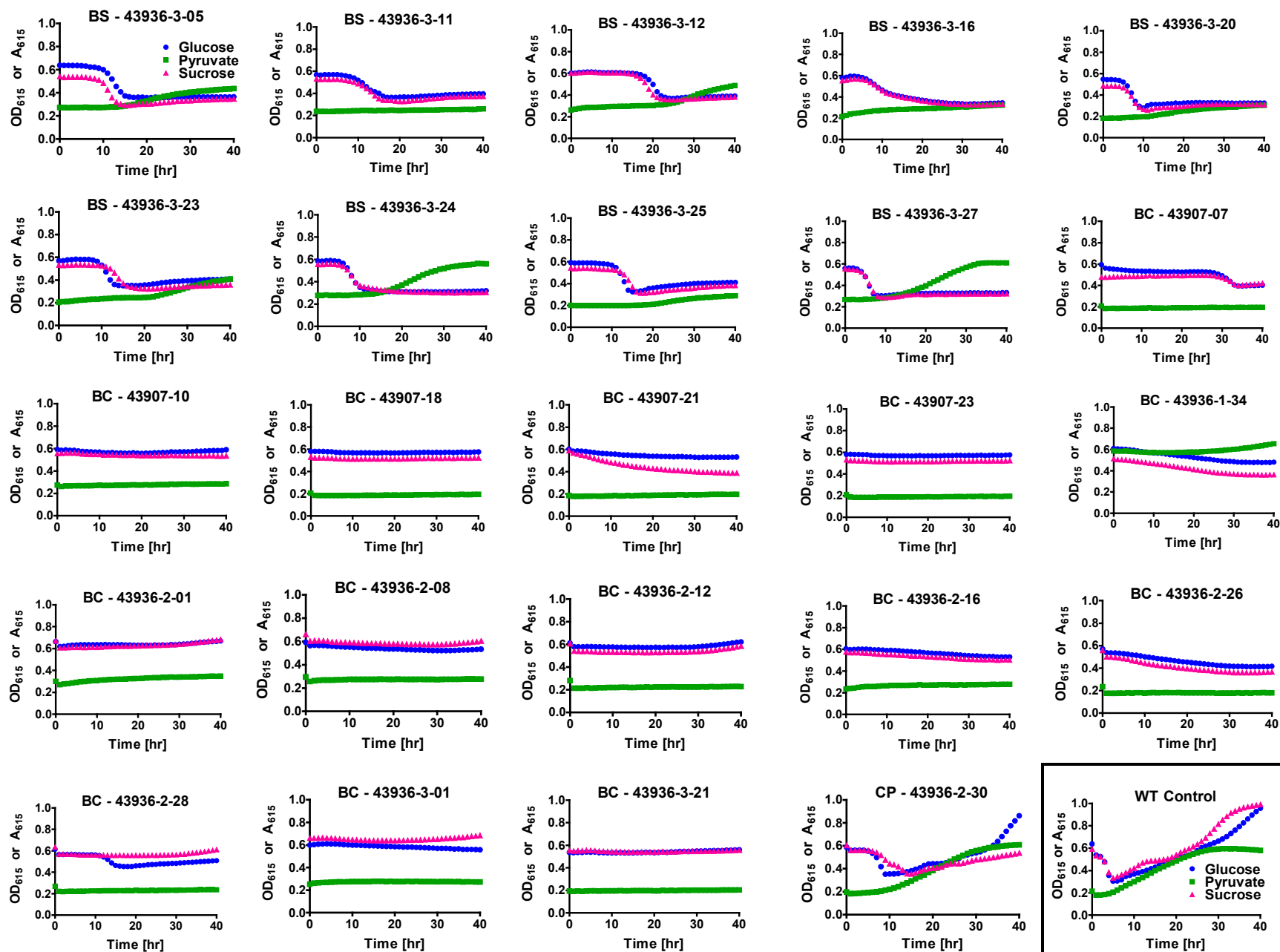

Supplement: Figure S1 — Natural extracts with reproducible effects on medium acidification by V. cholerae . Time course measurements of V. cholerae medium acidification in pH-MMSuc and pH-MMGlu or growth in MMPyr either alone (boxed trace) or in the presence of test extracts. Extract designation is indicated above each trace (see Table S1 for additional information). (PDF) [file pone.0031307.s001.pdf]

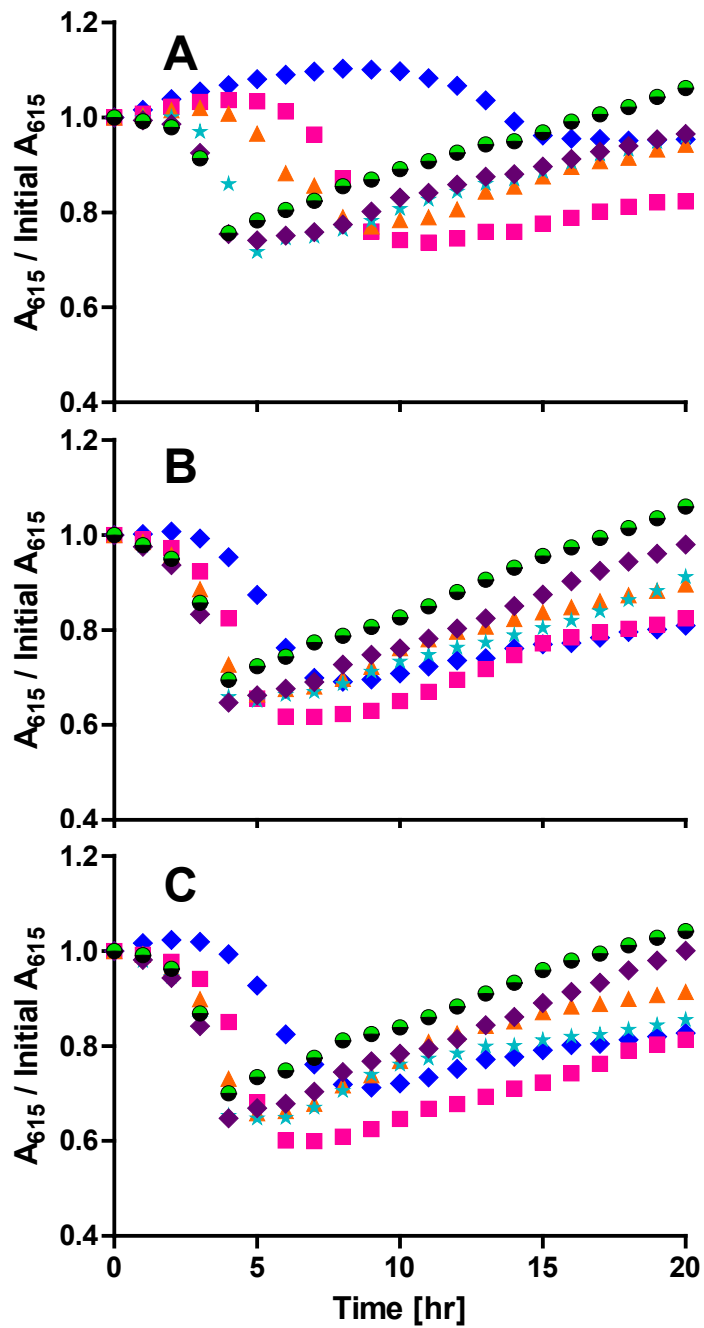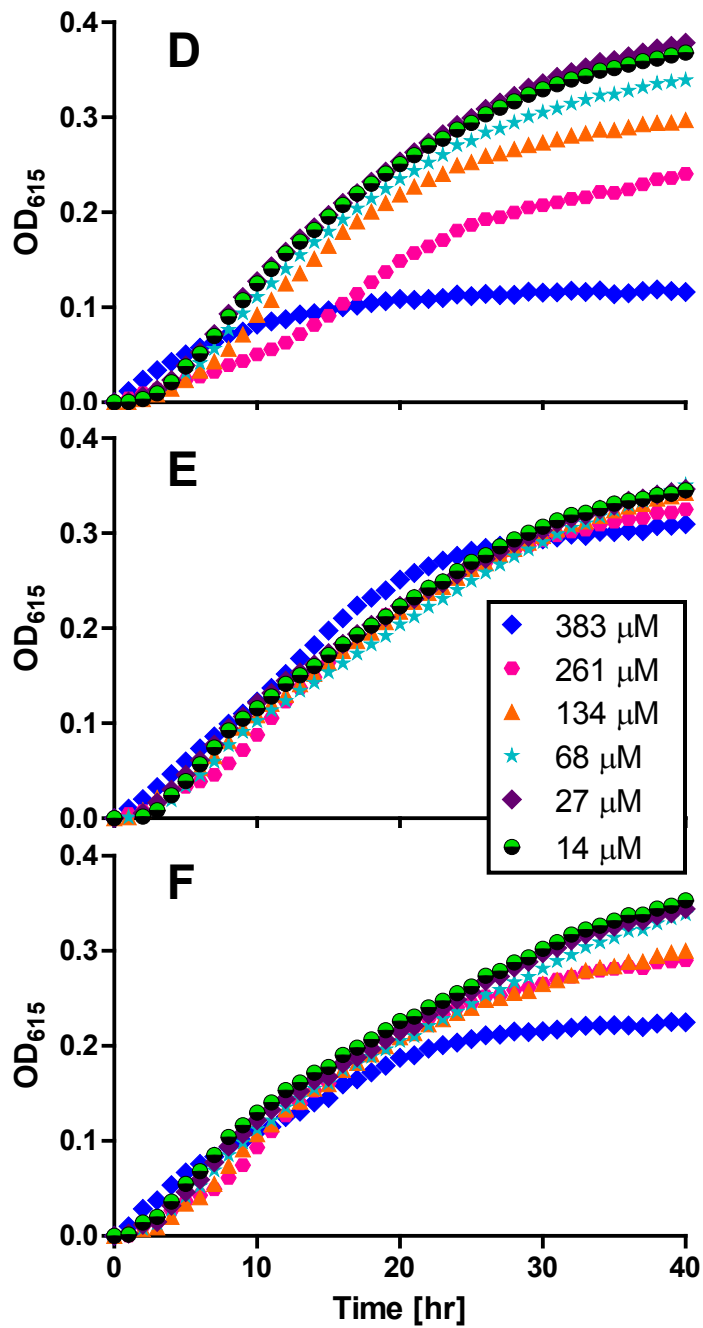

Supplement: Figure S2 — Impact of compounds 1, 2, and 3 on V. cholerae sugar fermentation and growth. Experimental replicate of Figure 5. (PDF) [file pone.0031307.s002.pdf]
