# Supplementary material for: A High-Throughput Screen Identifies a New Natural Product with Broad-Spectrum Antibacterial Activity
Source: PLoS One. 2012 Feb 16;7(2):e31307. doi: 10.1371/journal.pone.0031307 (PMC3281070; doi:10.1371/journal.pone.0031307)
Supplement: Table S1 — Natural extracts with reproducible effects on medium acidification by V. cholerae. (PDF) [file pone.0031307.s003.pdf]

Table S1: Natural extracts with reproducible effects on medium acidification by V. cholerae

Cherry Pick Run#	Plate	Well	Phenotype
BS-43936-1-01	1928	M22	bacteriostatic
BS-43936-1-02	1930	E15	bacteriostatic
BS-43936-1-03	1931	G09	bacteriostatic
BS-43936-1-06	1932	H13	bacteriostatic
BS-43936-1-14	1939	J22	bacteriostatic
BS-43936-1-22	1955	E05	bacteriostatic
BS-43936-1-23	1956	E12	bacteriostatic
BS-43936-1-24	1957	G01	bacteriostatic
BS-43936-1-26	1960	E01	bacteriostatic
BS-43936-1-28	1960	P04	bacteriostatic
BC-43936-1-34	1970	N01	bactericidal
BC-43936-2-01	1971	A17	bactericidal
BS-43879-11	1972	D15	bacteriostatic
BS-43936-2-05	1975	B20	bacteriostatic
BS-43936-2-06	1975	C21	bacteriostatic
BS-43936-2-07	1977	D15	bacteriostatic
BC-43936-2-08	1977	H02	bactericidal
BS-43936-2-10	1980	D10	bacteriostatic
BC-43936-2-12	1983	P05	bactericidal
BC-43936-2-16	1994	L22	bactericidal
BS-43936-2-22	2000	C13	bacteriostatic
BS-43936-2-24	2001	L12	bacteriostatic
BC-43907-07	2002	N19	bactericidal
BS-43879-23	2003	D06	bacteriostatic
BC-43936-2-26	2004	H22	bactericidal
BC-43936-2-28	2005	O01	bactericidal
CP-43936-2-30	2014	A05	differential sucrose and glucose fermentation
BS-43936-2-32	2015	M05	bacteriostatic
BS-43907-09	2016	A08	bacteriostatic
BC-43907-10	2018	H17	bactericidal
BS-43936-2-34	2018	J02	bacteriostatic
BC-43936-3-01	2019	M19	bactericidal
BS-43936-3-05	2028	D20	bacteriostatic
BS-43907-13	2028	F08	bacteriostatic
BS-43907-16	2038	F02	bacteriostatic
BS-43936-3-11	2041	A22	bacteriostatic
BS-43936-3-12	2041	B18	bacteriostatic
BS-43936-3-16	2041	J15	bacteriostatic
BC-43907-18	2042	A07	bactericidal
BS-43907-19	2044	A19	bacteriostatic
BS-43907-20	2045	O09	bacteriostatic
BC-43907-21	2046	J09	bactericidal
BC-43907-23	2049	K06	bactericidal
BS-43936-3-20	2049	O16	bacteriostatic
BC-43936-3-21	2053	C01	bactericidal
BS-43936-3-23	2053	E01	bacteriostatic
BS-43936-3-25	2053	J06	bacteriostatic
BS-43936-3-24	2053	M17	bacteriostatic

BS-43936-3-27	2054	O07	bacteriostatic
