# Supplementary material for: A High-Throughput Screen Identifies a New Natural Product with Broad-Spectrum Antibacterial Activity
Source: PLoS One. 2012 Feb 16;7(2):e31307. doi: 10.1371/journal.pone.0031307 (PMC3281070; doi:10.1371/journal.pone.0031307)
Supplement: Table S2 — 1H and 13C NMR data of compounds 1 to 3. (PDF) [file pone.0031307.s004.pdf]

Table S2. ^{1}H[a] and ^{13}C[b] NMR Data of Compounds **1** to **3** (in CD_3OD)

	^{1}H			^{13}C		
#	**1** (SC2-133-1)	**2** (SC2-133-2)	**3** (SC2-133-3)	**1**	**2**	**3**
1				149.9	153.5	189.2
2				131.4	128.1	147.6
3				121.4	124.6	141.9
4				148.9	150.2	188.4
5	6.56 (d, 8)	6.52 (d, 8)	6.77 (br s)	115.4	114.9	137.4
6	6.48 (d, 8)	6.43 (d, 8)	6.77 (br s)	114.0	108.9	137.4
7	2.60 (m)	3.34 (dd, 12, 6)<br>2.80 (dd, 18, 6)	2.55 (m)	28.7	36.9	28.8
8	1.51 (m)	4.82 (m)	1.48 (m)	24.1	80.5	24.1
9	0.96 (t, 6.6)	1.39 (t, 6.6)	0.97 (t, 6.6)	14.2	21.7	14.4
10	4.52 (s)	4.61 (s)	4.47 (s)	55.5	58.6	55.3

[a]δ (ppm) 600 MHz; multiplicities; *J* values (Hz) in parentheses. [b]δ (ppm) 150 MHz, from gHSQCed and gHMBC.

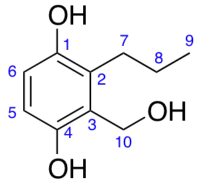

1

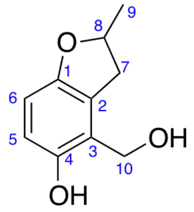

2

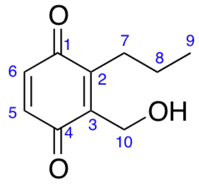

3

In the COSY spectrum of **1**, two spin systems were observed, H-H and CH_2-CH_2-CH_3. In the HMBC spectrum of **1**, ^2J and ^3J correlations from H-7 to C-1 (δ_C 149.9), C-2, and C-3, and from H-10 to C-2, C-3, and C-4 (δ_C 148.9) were observed indicating that **1** was a hydroquinone with the propyl and hydroxymethyl groups adjacent to each other, and the two aromatic protons at the other side of the molecule. Hence, the structure of **1** was determined as shown. Similarly, the structures of compounds **2** and **3** were also elucidated using spectroscopic data.
