# Supplementary material for: A High-Throughput Screen Identifies a New Natural Product with Broad-Spectrum Antibacterial Activity
Source: PLoS One. 2012 Feb 16;7(2):e31307. doi: 10.1371/journal.pone.0031307 (PMC3281070; doi:10.1371/journal.pone.0031307)
Supplement: Text S1 — Sequence analysis of products obtained from amplification of the internal transcribed spacer of isolate 1223-D. (PDF) [file pone.0031307.s005.pdf]

>LR5 as 5’ primer

TCGATTAGTCTTTCGCCCCCATGCCCATATTTGACGATCGATTTGCACGTCAGAAC

CGCTGCGAGCCTCCACCAGAGTTTCCTCTGGCTTCACCCTATACAGGCATAGTTCA

CCTTCTTTCGGGTCCGGCCCCGTATGCTCTTACTCAAATCCATCCGAGAACATCAG

GATCGGTCGGAGATGCGCCGAAGCTCTCTCCTACGTTCACTTTCATTACGCGTAG

GGGTTTGACACCCGAACACTCGCATACGAAGACGACTCCTTGGTCCGTGTTTCAA

GACGGGTCGCTGATGACCATTACGCCAGCATCCTTGCAGAAGCGCGAACCTCAGT

CGACCCCAGGGTATTACGCAGCGGGCTATAACACTCCCGGAGGAGCCACATTCCC

GAAGCCTTTATCCCCCGGGGCCAACTGATGCTGGCCTGAGCCGGCAGAGTGCAC

CACCGAGAACGATGGATGATCAACCGGCCCAAGTCTGGTCATGAGCGCTTCCCTT

TCAACAATTTCACGTACTGTTTAACCCTCTTTTCAAAGTGCTTTTCATCTTTCGATCA

CTCTACTTGTGCGCTATCGGTCTCTGGCCGGTATTTAGCTTTAGAAGACGTATACC

TCCCATTTAGAGCAGCATTCCCAAACTACTCGACTCGTCGAAGGAGTTTCACAGAG

GCTTAGCGACCAACCGTACGGGGCTCTCACCCTCTATGGCGTCCCGTTCCAGGGA

ACTCGGAAGGCACCTCGCCAGNNCATCCTCTGCAAATTACAACTCGGGCCGGGG

GCCAGATTTCAAATTTGAGCTGTTGCCGCTTCACTCGCCGTTACTGAGGCAATCCC

TGTTGGTTTCTTTTCCTCCGCTTATTGATATGCTA

>LROR as 5’ primer

TGCCTCAGTAACGGCGAGTGAAGCGGCAACAGCTCAAATTTGAAATCTGGCCCCC

GGCCCGAGTTGTAATTTGCAGAGGATGTTTCTGGCGAGGTGCCTTCCGAGTTCCC

TGGAACGGGACGCCATAGAGGGTGAGAGCCCCGTACGGTTGGTCGCTAAGCCTC

TGTGAAACTCCTTCGACGAGTCGAGTAGTTTGGGAATGCTGCTCTAAATGGGAGGT

ATACGTCTTCTAAAGCTAAATACCGGCCAGAGACCGATAGCGCACAAGTAGAGTGA

TCGAAAGATGAAAAGCACTTTGAAAAGAGGGTTAAACAGTACGTGAAATTGTTGAA

AGGGAAGCGCTCATGACCAGACTTGGGCCGGTTGATCATCCATCGTTCTCGGTGG

TGCACTCTGCCGGCTCAGGCCAGCATCAGTTGGCCCCGGGGGATAAAGGCTTCG

GGAATGTGGCTCCTCCGGGAGTGTTATAGCCCGCTGCGTAATACCCTGGGGTCGA

CTGAGGTTCGCGCTTCTGCAAGGATGCTGGCGTAATGGTCATCAGCGACCCGTCT

TGAAACACGGACCAAGGAGTCGTCTTCGTATGCGAGTGTTCGGGTGTCAAACCCC

TACGCGTAATGAAAGTGAACGTAGGAGAGAGCTTCGGCGCATCTCCGACCGATCC

TGATGTTCTCGGATGGATTTGAGTAAGAGCATACGGGGCCGGACCCGAAAGAAGG

TGAACTATGCCTGTATAGGGTGAAGCCAGAGGAAACTCTGGTGGAGGCTCGCAGC

GGTTCTGACGTGCAAATCGATCGTCAAATATGGGGCATGGGGGGCGAAAGACTAA

TCGAACCTTCTAGTAGCTGGTTTCCGC
