# Supplementary material for: A High-Throughput Screen Identifies a New Natural Product with Broad-Spectrum Antibacterial Activity
Source: PLoS One. 2012 Feb 16;7(2):e31307. doi: 10.1371/journal.pone.0031307 (PMC3281070; doi:10.1371/journal.pone.0031307)
Supplement: Text S2 — Physical characterization of compounds 1, 2, and 3 derived from CR1223-D. (PDF) [file pone.0031307.s006.pdf]

SC2-133-1 (compound 1): light yellow powder; UV (MeOH) λ_{max} (log ε) 207 (4.16), 299 (3.48) nm; IR ν 3327, 2961, 2931, 2872, 1648, 1585, 1487, 1464, 1349, 1282, 1254, 1224, 1102, 998, 968, 811, 758 cm^{-1}; ^{1}H NMR (600 MHz, CD_3OD); see Table S2; ^{13}C NMR (150 MHz, CD_3OD): see Table S2; HRMS *m/z* 205.0839 ([M+Na], calcd for $C_{10}H_{14}O_3Na$, 205.0841).

SC2-133-2 (compound 2): light yellow powder; $[a]^{23}_{D}$ +2.1 (*c* 0.28, EtOH); UV (MeOH) λ_{max} (log ε) 206 (3.80), 223 (sh), 235 (sh), 305 (3.03) nm; IR ν 3323, 2963, 2918, 1646, 1579, 1462, 1380, 1348, 1227, 1028, 989, 813, 767 cm^{-1}; ^{1}H NMR (600 MHz, CD_3OD); see Table S2; ^{13}C NMR (150 MHz, CD_3OD): see Table S2; HRMS *m/z* 203.0686 ([M+Na], calcd for $C_{10}H_{12}O_3Na$, 203.0684).

SC2-133-3 (compound 3): yellow powder; UV (MeOH) λ_{max} (log ε) 204 (3.55), 247 (3.18), 300 (2.71), 381 (2.28) nm; IR ν 3336, 2962, 2926, 1647, 1587, 1464, 1384, 1350, 1280, 1101, 1010 cm^{-1}; ^{1}H NMR (600 MHz, CD_3OD); see □Table S2; ^{13}C NMR (150 MHz, CD_3OD): see Table S2; HRMS *m/z* 203.0681 ([M+Na], calcd for $C_{10}H_{12}O_3Na$, 203.0684).

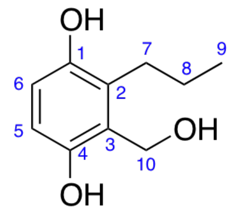

1

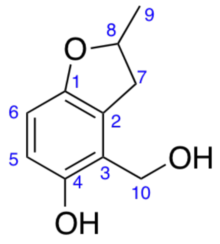

2

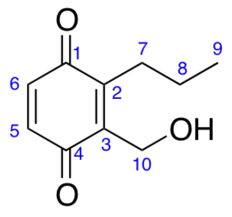

3
